# From therapeutic to elective cesarean deliveries: factors associated with the increase in cesarean deliveries in Chiapas

**DOI:** 10.1186/s12939-017-0582-2

**Published:** 2017-05-25

**Authors:** María Graciela Freyermuth, José Alberto Muños, María del Pilar Ochoa

**Affiliations:** 1 The Centro de Investigaciones y Estudios Superiores en Antropología Social (CIESAS), Unidad Sureste and Technical Secretary of the Observatory of Maternal Mortality in Mexico (OMM), San Cristobal de las Casas, Chiapas México; 2 The CONACYT- Center of Research and Higher Studies in Social Anthropology (CIESAS), South Pacific Unit, Oaxaca, México; 3 Masters in Population and Development, Advisor in the Ministry of Health, Ciudad de México, México

**Keywords:** Cesarean, Medicalization, Chiapas, Indigenous population, Maternal mortality

## Abstract

**Background:**

Cesarean deliveries have increased over the past decade in Mexico, including those states with high percentages of indigenous language speakers, e.g., Chiapas. However, the factors contributing to this trend and whether they affect indigenous languages populations remain unknown. Thus, this work aims to identify some of the factors controlling the prevalence of cesarean sections (C-sections) in Chiapas between the 2011–2014 period.

**Methods:**

We analyzed certified birth data, compiled by the Subsystem of Information on Births of the Secretary of Health and the National Institute of Statistics and Geography, and information regarding the Human Development Index (HDI), assembled by the United Nations Development Program. A descriptive analysis of the variables and a multilevel logistics regression model were employed to assess the role of the different factors in the observed trends.

**Results:**

The results show that the factors contributing to the increased risk of C-sections are (i) women residing in municipalities with indigenous population and municipalities with high HDIs, (ii) advanced schooling, (iii) frequent prenatal checkups, and (iv) deliveries occurring in private health clinics. Furthermore, C-sections might also be associated with prolonged hospital stays.

**Conclusions:**

The increasing frequency of C-sections among indigenous populations in Chiapas seems to be related to public policies aimed at reducing maternal mortality in Mexico. Therefore, public health policy needs to be revisited to ensure that reproductive rights are being respected.

## Background

In 1922, J. Whitridge Williams declared, “the excellence of an obstetrician should be gauged not by the number of cesareans which he performs, but rather by those he does not do.”^1^ In the 1980s, health professionals believed that the rate of cesarean deliveries should be 10–15% [1]. Regardless of the region, healthcare professionals consider higher rates to be unjustified in light of the frequency of obstetric complications [2–4]. Nevertheless, cesarean sections (C-sections) are among the most common surgeries performed, with increasing frequencies being observed in developed and developing countries [5–9].

**Fig. 1:**
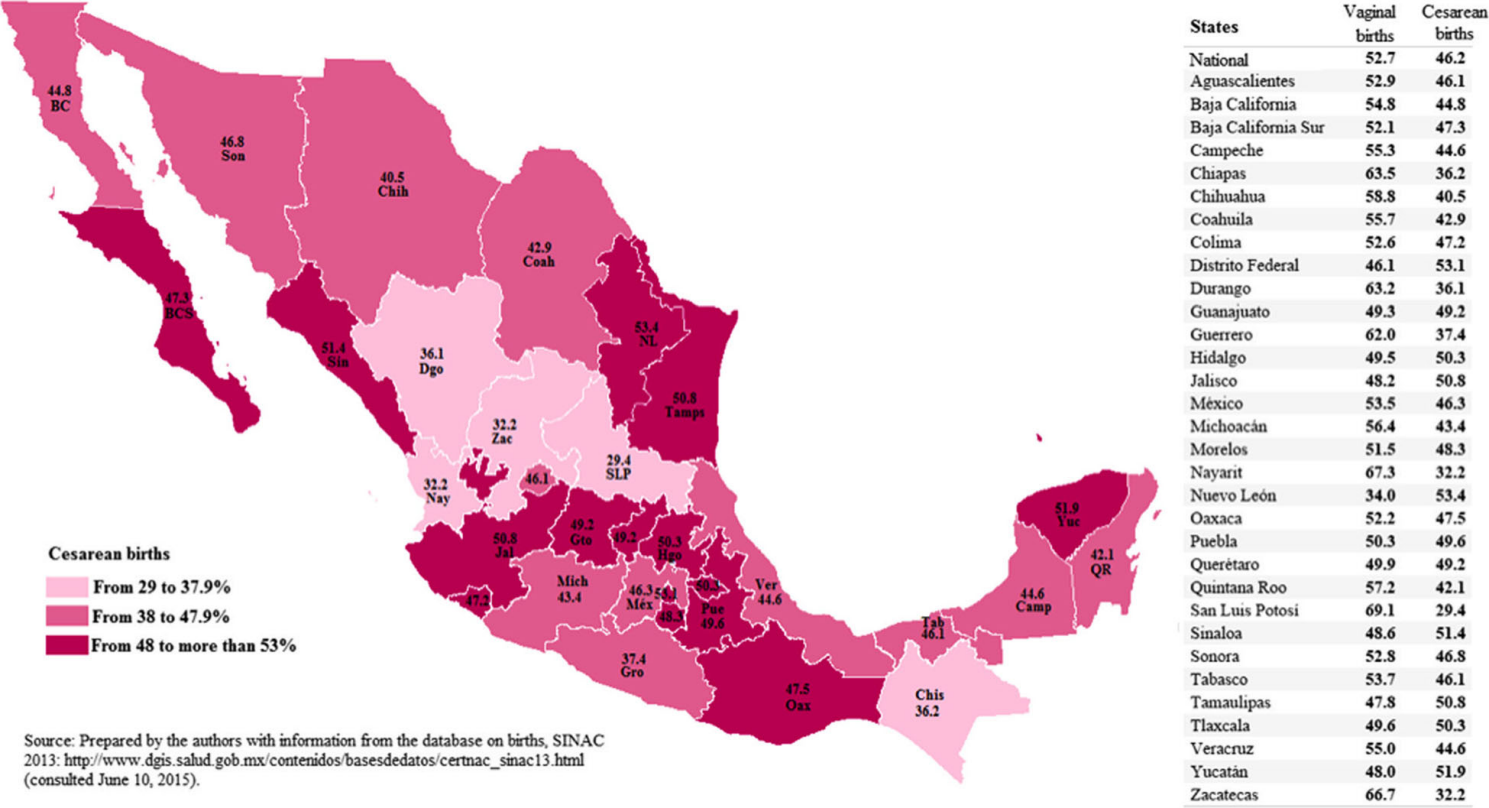
Cesarean births, by state, in Mexico, 2013 (in %)

Over the past decade, throughout Mexico, there has been a significant increase in the percentage of cesarean deliveries, even in states with high percentages of speakers of indigenous languages (SILs), such as Chiapas (Fig. 1). According to a National Survey of Health and Nutrition comparative analysis, undertaken in 2000, 2006, and 2012, the number of C-sections performed in Mexico increased by 50% over a 12-year period, associated to an increase of 33% and 66% in the public and private sectors, respectively [10]. Nationally, between 2011 and 2014, there were 8,746,144 births, with almost half (4,012,894) involving C-sections. The Mexican state of Chiapas has experienced a gradual increase in the proportion of the population having medical insurance and an increase in the number of C-sections. In 2002, 13% of registered live births^2^ involved cesarean delivery, whereas in 2012 that figure increased to 32%, representing an increase of almost 120% over a 10-year period [11].

In recent studies, the factors influencing the increase in C-sections, in Mexico, include those associated with the mother, physician, and the situational context. Puentes et al. [12] reported that the type of institution in which delivery occurs, the mother’s educational level, the number of physicians per capita, and the Human Development Index are factors influencing the rising numbers of C-sections. Furthermore, Suárez et al. [13] stated that age, socioeconomic status, place of residence, parity, number of previous abortions, and total numbers of prenatal checkups are associated with the increasing number of C-sections.

Martínez et al. [14] analyzed the diagnoses contributing to decisions to perform C-sections, and found that obstetrician attitudes are not based on specific medical tests, but on cultural factors, legal responsibilities, and perinatal care variables [14]. Accordingly, Vallejos et al. [15] used a perception instrument to analyze the physicians’ regarding C-sections. They identified a tendency of physicians to respond in a predetermined way to a particular stimulus [15]. A study conducted in 2012 at a Mexican Social Security Institute (Instituto Mexicano del Seguro Social, IMSS)-affiliated hospital in Yucatan, showed that the largest proportion of C-sections was performed in primiparous women and in women 21–25-years-old who had severe preeclampsia as the main cause of surgical intervention [16].

Juarez et al. [17] showed, based on administrative births between 1991 and 1995 that increases in C-section rates are higher among Mexican women affiliated with social security institutions. Moreover, the larger increases corresponded with the states having greater levels of economic development, such as Nuevo Leon, Federal District, and the State of Mexico, compared with less developed states, such as Chiapas, Guerrero, and Oaxaca [17].

In Chiapas, a 1998 survey showed that >95% of the population lacked access to social security and that 2% of indigenous women underwent C-sections; in contrast, the rate among non-indigenous women was 7.2%. Among women receiving medical care during delivery, 14.7% were indigenous and 24.3% were non-indigenous. In addition to the significant difference in cesarean rates, the lack of access to health services was notable, for both groups. In 2008, an evaluation performed for the IMSS Oportunidades program (National Institute of Public Health) indicated that among rural hospitals, two from Chiapas (along with eight others) had the poorest results in all the indicators that comprise them, and received recommendations on prioritizing their actions in deliveries of women under the age of 20, detection of pregnancies during the first trimester, C-sections, in-hospital mortality, daily consultations and surgeries [18].

Nazar et al. performed a historical review of 1993–2002 birth records, and reported that the rate of cesarean deliveries among indigenous women increased from 0% to 10%, and among non-indigenous women from 2% to 25% for such time period [19].

Prior studies have focused on identifying the diagnoses leading to C-sections and their prevalence in hospitals for indigenous and non-indigenous women. For instance, a study conducted in Chile showed that the prevalence of C-sections among Aymara and non-Aymara populations were 27.6% and 26.7%, respectively [20]. Aymara women were likely to have C-sections due to fetal distress and cephalopelvic disproportion, whereas C-sections among non-Aymara women were predominantly due to stationary dilatation, dystocia, and poor obstetric condition [20].

Studies in Chiapas showed a direct relationship between delivery medicalization and increased frequencies of C-sections. The remarkable growth in the prevalence of C-sections, in Chiapas, began in 2004 when the Popular Health Insurance program was initiated. In 2000, only 17% of women in Chiapas had social security or other types of health coverage.^3^ In 2004, when the Seguro Popular (SP) began accepting members, 20% of women had some type of medical coverage. Of the female health coverage beneficiaries, almost a quarter (23%) used SP, a striking difference from women in Mexico City, where only 4% of beneficiaries were SP enrollees.^4^

In 2008, the “Healthy Pregnancy” program was launched in Mexico, leading to the immediate affiliation of pregnant women with SP. By 2010, 100% of the eligible population was enrolled in SP, according to the Fourth Annual Report of the Government of Chiapas [21]. However, in 2014, the number of women in Chiapas enrolled with SP was 1,923,702, i.e., 92% of the goal set for this program over the government’s 6-year term (2012–2018) [22]. In 2002, during the period of growing affiliation, 67% of the projected number of births in Chiapas did not occur in health centers. By 2012, this number had decreased to 40%. In one decade, the frequency of institutional childbirth and the number of cesarean deliveries both increased by almost 100%.^5^

During the first half of the 20th century, C-sections were a therapeutic procedure for very precise reasons: premature detachment of the placenta, failed induction of labor, prior C-section, severe pre-eclampsia/eclampsia, prolapsed umbilical cord, fetal distress, breech delivery/presentation other than head down, cephalopelvic disproportion/prolonged labor, multiple pregnancy, vesicovaginal fistula, and, more recently, presence of maternal viral illnesses (human immunodeficiency virus, human papillomavirus). In the mid-20th century, cesarean deliveries were firmly embedded in obstetric techniques, leading to a drop in maternal deaths; later, however, the procedure spiraled out of control and was used without medical justification [22–26].

Fetal distress, dystocia, breech birth, and previous cesarean deliveries made up 90% of the reasons for performing this treatment, with some arguing in favor of wider use owing to the procedure’s relatively low maternal and neonatal morbidity and mortality [27]. However, unnecessary C-sections can present important drawbacks for either mother or newborns [28]. One of the principal risk factors associated to C-sections is the surgical wound infection, which may lead to multiple complications [29, 30]. Furthermore, uterine rupture can lead to vesicovaginal fistula formation [31] and to deformations that can cause infertility [32]. C-sections are also considered to increase the risk of hemorrhages because they cause placenta previa, placenta accreta, premature detachment of the placenta, and uterine rupture [33, 34] in subsequent pregnancies. Post-cesarean morbidity also affects newborns, given its links to several types of respiratory complications [32]. In addition, changes in the degree of methylation of specific gene deoxyribonucleic acid (DNA) in descendants are associated with the number of hours in labor [35]. A higher degree of DNA methylation was observed in babies born to mothers who had a cesarean delivery, and this alteration might affect their immune responses [36]. On the other hand, some authors have detected a positive association between post-cesarean antibiotic treatment and severe maternal morbidity and mortality, as well as a direct association between cesarean rates and fetal death rates [37]. Therefore, it is important to assess the factors controlling the prevalence if C-sections.

To the best of the authors’ knowledge, previous studies have not documented the sociodemographic characteristics of women that contribute to the increased C-section frequency in Chiapas. Thus, the aim of this study is twofold. Firstly, to describe and analyze the prevalence of cesarean births in Chiapas between 2011 and 2014. Secondly, to identify the factors contributing to the increased frequency of C-sections. We consider C-sections to be a medical practice that is associated with various structural, institutional, and personal causes.

## Methods

We performed a retrospective, crosscut, descriptive, observational study to analyze cesarean births in Chiapas from 2011 to 2014 employing different databases and a statistical analysis.

### Information sources

The data source employed in this study was primarily the Subsystem of Information on Births in the Ministry of Health (known in Spanish as SINAC). This database arises from the establishment in Mexico of birth certificate in 2007. Nevertheless, it was not until 2011 when the SSA proposed to use this data for calculation of sociodemographic indicators such as maternal mortality, infant mortality, and fertility since they were not considered reliable until that date.

Throughout Mexico, this source gathers information on live births and the conditions under which they occur. In Chiapas, underreporting and other inaccuracies in these administrative records are likely. However, we relied on this data source because it takes these problems into account and uses adjustment factors to validate information quality. Because a greater quantity of data minimizes errors and renders data more consistent [38], our review of birth records in Chiapas, between 2011 and 2014, examined 349,058 certified births.

Another source of information was the United Nations Development Program (UNDP), which compiled the Human Development Index (HDI),^6^ by municipality, in 2010. Finally, we used the percentage of SILs, by municipality, with categories taken from the birth statistics databases of the Dirección General de Información en Salud (DGIS), 2013.

### Variable selection

We compiled a 14-variable database that included birth order, maternal age and schooling, municipality of residence (HDI and percentage of SILs), total numbers of prenatal checkups, trimester of first prenatal checkup, birthing facility, maternal marital status, maternal health affiliation program, multiple pregnancy, newborn weight, gestational age, and hospitalization duration. Some inconsistencies between the age and schooling level variables were found, resulting in 818 cases being discarded.

One municipal variable incorporated into the analysis was the 2010 HDI, by municipality, obtained from UNDP data [39]. We included this index because it reflects the advances or setbacks in people’s ability to exercise their basic rights in health, income, and education. The four groups established by UNDP (low, medium-low, medium-high, and high) were encoded.

A second external variable included in the database was the percentage of SILs, by municipality, with categories taken from the DGIS birth records [40]; 27% of the population of Chiapas, ≥3-years-old, is an SIL [41]. Thus, this characteristic was believed to potentially differentiate access to health services and, therefore, influence the type of birth procedure. The SIL variable was encoded into three categories: indigenous municipality (≥40% indigenous population), municipality with the presence of an indigenous population (<40% indigenous population and >5000 indigenous inhabitants), and municipalities with a dispersed indigenous population (<40% indigenous population and <5000 indigenous inhabitants.)

### Statistical analysis

Once the database was compiled, descriptive statistics (percentage distributions and frequencies) were calculated to describe the prevalence of C-sections and the sociodemographic characteristics of the women, the childbirth care institutions, and the newborns.

A multilevel mixed-effects logistic regression analysis [42] was then conducted. We used this type of model because a hierarchical structure, with respect to the municipal variable, was detected. Thus, for this data type, the responses or characteristics of individuals belonging to the same group are generally more similar than those between groups. This implies a potential distortion in the group effects and may lead to interpretative fallacy when the coefficients that report on aggregated behaviors are used to make inferences at the individual level. Similarly, atomistic fallacy may occur when making inferences about the groups by analyzing the aggregate units [43]. To address this problem, we used a mixed logistics model with fixed covariable effects and random effects for the groups. This type of model allowed us to assess the extent that the analyzed variables or factors increased or decreased the cesarean birth possibility. To this end, we dichotomized the response variable analyzed by assigning cesarean births a value of “1” and vaginal births a value of “0”.

Based on the results of a literature review regarding cesarean deliveries, and the results of the descriptive analysis, we chose the following explanatory variables: birth order (first or second, third to fifth, sixth to tenth, eleventh or greater), maternal marital status (married at any time,^7^ married, single); maternal schooling level (none or did not finish primary school, finished primary school, finished secondary school, finished high school, university or post-graduate); maternal age (<17, 18–24, 25–34, 35–44, ≥45 years); maternal health affiliation program (none, IMSS,^8^ Instituto de Seguridad y Servicios Sociales para los Trabajadores del Estado [ISSSTE],^9^ Servicios de Salud de Petróleos Mexicanos [PEMEX],^10^ Servicios de Salud de la Secretaría de la Defensa Nacional [SEDENA] or Servicios de Salud de la Secretaría de Marina-Armada de México [SEMAR],^11^ SP,^12^ IMSS-Oportunidades,^13^ other); trimester of first prenatal checkup (no checkup, first, second, third); total number of prenatal checkups (0–1, 2–5, ≥6); multiple pregnancy (one, multiple); newborn weight (<2500 g, 2501–3999 g, ≥4000 g); hospitalization duration (0–1, 2–7, 8–30, 31–99, ≥100 days); gestational age (<28, 28–36, 37–41, and 42–45 weeks); birth facility (SSA^14^; IMSS-Oportunidades; IMSS; ISSSTE; PEMEX, SEDENA, or SEMAR; another public facility; private medical facility); indigenous presence (indigenous population, population with indigenous presence, dispersed indigenous population); and HDI (low, medium-low, medium-high, high).

The total number of observations in this model was 190,763. To encode the independent variables, we discarded “not specified” or “does not know” answers. The analysis was performed using STATA 11 (StataCorp, College Station, TX, USA); we used the *xtmelogit* command to calculate a multilevel mixed-effects logistic regression for models with binary/binomial responses.

## Results

The results of the descriptive analysis (Table 1) show that approximately 40% of the 349,058 certified births, during the 2011–2014 period, corresponded to women who had their first or second child by C-section, i.e., approximately 9% less than the national figure of 49%. A frequency analysis revealed that C-sections occurred more frequently for the first or second pregnancy, and decreased as the total number of pregnancies increased.

**Table 1:** Factors associated with cesarean births in Chiapas, 2011–2014

Characteristics	n	Type of birth (%)		95% Confidence interval	
		Vaginal	Cesarean	Lower	Upper
Type of delivery	349,058	64.4	35.6	0.35	0.36
Order of birth (*n* = 346,789)					
First or second	218,912	59.6	40.4	0.40	0.41
Third to fifth	108,187	71.1	28.9	0.29	0.29
Sixth to tenth	18,533	83.6	16.4	0.16	0.17
Eleventh or greater	1157	82.5	17.6	0.15	0.20
Maternal age, y (*n* = 345,740)					
< 17	27,790	70.5	29.5	0.29	0.30
18–24	145,033	67.5	32.5	0.32	0.33
25–34	139,167	61.2	38.8	0.39	0.39
35–44	32,895	58.5	41.5	0.41	0.42
≥ 45	855	70.2	29.8	0.27	0.33
Maternal level of schooling (*n* = 342,768)					
None or did not finish primary school	74,998	77.0	23.0	0.23	0.23
Finished primary school	87,993	71.6	28.4	0.28	0.29
Finished secondary school	96,953	65.4	34.6	0.34	0.35
Finished high school	52,171	54.0	46.1	0.46	0.46
University or postgraduate degree	30,653	28.4	71.6	0.71	0.72
Total prenatal checkups (*n* = 329,384)					
0–1	31,422	78.7	21.4	0.21	0.22
2–5	98,853	71.2	28.9	0.29	0.29
≥ 6	199,109	59.1	40.9	0.41	0.41
Trimester of first prenatal checkup (*n* = 338,693)					
None	24,323	77	23	0.22	0.24
First	218,463	60	40	0.40	0.40
Second	79,090	71	29	0.29	0.29
Third	16,817	71	29	0.28	0.30
Facility where birth occurred (*n* = 317,286)					
Secretary of Health	188,792	66.3	33.7	0.33	0.34
IMSS-Oportunidades	61,539	76.8	23.2	0.23	0.24
IMSS	25,579	50	50	0.49	0.51
ISSSTE	4373	30.1	69.9	0.69	0.71
PEMEX, SEDENA, or SEMAR	2228	60.2	39.8	0.38	0.42
Other public facility	2867	27.8	72.2	0.71	0.74
Private medical facility	31,908	13.9	86.1	0.86	0.87
Indigenous presence (*n* = 349,058)					
Indigenous population	97,410	75.3	24.7	0.24	0.25
Population with indigenous presence	187,040	57.5	42.5	0.42	0.43
Dispersed indigenous population	64,608	68.6	31.9	0.31	0.32
Human Development Index (*n* = 349,023)					
Low	2,241	97.6	2.4	0.02	0.03
Medium low	129,155	81.2	18.8	0.19	0.19
Medium high	145,944	55.2	44.8	0.45	0.45
High	71,683	51.9	48.1	0.48	0.48
Maternal marital status (*n* = 340,386)					
Married at any time	2152	67.8	32.2	0.30	0.34
Married	318,360	64.8	35.2	0.35	0.35
Single	19,874	60	40	0.39	0.40
Maternal health affiliation program (*n* = 327,705)					
None	65,822	70.3	29.7	0.29	0.30
IMSS	31,194	44.8	55.2	0.54	0.56
ISSSTE	6597	25.4	74.6	0.73	0.76
PEMEX, SEDENA or SEMAR	2497	58.9	41.1	0.39	0.43
SP	208,421	67	33	0.32	0.33
IMSS-Oportunidades	9028	85.4	14.7	0.14	0.15
Other	4146	26.1	73.9	0.72	0.75
Multiple pregnancies (*n* = 348,210)					
One	343,276	64.9	35.1	0.35	0.35
Multiple	4934	29.7	70.3	0.69	0.71
Weight of newborn, g (*n* = 327,613)					
< 2,500 g	18,625	48.2	51.8	0.51	0.52
2501–3999	297,873	65.4	34.6	0.34	0.35
≥ 4000	11,115	59.9	40.1	0.39	0.41
Gestational age, weeks (*n* = 345,148)					
< 28	678	70.7	29.4	0.26	0.33
28–36	15,999	40.9	59.1	0.58	0.60
37–41	324,824	65.5	34.5	0.34	0.35
42–45	3647	43.7	56.3	0.55	0.58
Hospitalization period, days (*n* = 266,933)					
0–1	119,804	65.4	34.6	0.34	0.35
2–7	44,554	59.7	40.3	0.40	0.41
8–30	52,285	64.5	35.5	0.35	0.36
31–99	30,091	65.0	35.0	0.34	0.35
≥ 100	20,199	62.7	37.3	0.36	0.38

*IMSS* Instituto Mexicano del Seguro Social, *ISSSTE* Instituto de Seguridad y Servicios Sociales para los Trabajadores del Estado, *PEMEX* Servicios de Salud de Petróleos Mexicanos, *SEDENA* Servicios de Salud de la Secretaría de la Defensa Nacional, *SEMAR* Servicios de Salud de la Secretaría de Marina-Armada de México, *IMSS-Oportunidades* Instituto Mexicano del Seguro Social – Oportunidades (now known as Prospera)

The totals for each category differ because not all of the 349,058 births were associated with valid responses to each variable category, i.e., there were “unknown” and “unspecified” responses that were omitted

Source: Prepared by the authors with information from DGIS, Subsystem of Information on Births 2011–2014

Over the study period, the frequency of women having C-sections varied according to the maternal age: <18-years-old (30% had C-sections), 18–24-years-old (33%), 25–34-years-old (39%), 35–44-years-old (42%), and ≥45-years-old (30%). There was also a direct relationship between cesarean births and the mother’s level of schooling: 72% of women with university or graduate degrees had C-sections, whereas 23% of women who did not attend school or did not finish primary school had C-sections.

Only 21% of women who had one or fewer prenatal checkups had C-sections, whereas 41% of those who had ≥6 checkups had C-sections. Women who began prenatal checkups during the first trimester of their pregnancy had a higher C-section frequency than did those who began later (40% and 29%, respectively); C-sections accounted for 23% of deliveries for women not receiving prenatal care.

The percentage of cesarean births reached 86% among women attending private medical facilities, whereas it was 23% in IMSS-Oportunidades (now known as Prospera) facilities.

Women residing in municipalities with an indigenous presence had a C-section frequency of 43%, compared with 32% of women in municipalities with dispersed indigenous population and 25% of women in municipalities with an indigenous population. Among women who registered their child’s birth in Chiapas municipalities having lower HDIs, 2.4% had C-sections; that number rose to 48.1% among women giving birth in municipalities with higher HDIs.

### Multilevel logistics model results

This section shows the results obtained using multilevel mixed-effects logistic modeling, which indicates the possibility of a cesarean birth being associated with an explicative variable.

Table 2 shows the results obtained using the model. Women who had 3–5 prior childbirths had a 42% lower possibility of having a C-section than those delivering their first or second child. This decreased further for women who had 6–10 prior deliveries (64% fewer C-sections) than for the women in the reference group (those having their first or second delivery); compared to the reference group, women who had ≥11 prior deliveries were 54% less likely to have C-sections.

**Table 2:** Multivariate logistical regression of factors associated with cesarean births in Chiapas, 2011–2014

Cesarean (1 = yes, 0 = no)	Exp (B)	P > z	95% Confidence interval	
			Lower	Upper
Order of birth				
First or second	−	−	−	−
Third to fifth	0.580^a^	0	0.56	0.60
Sixth to tenth	0.365^a^	0	0.34	0.39
Eleventh or higher	0.457^a^	0	0.36	0.58
Maternal level of schooling				
None or did not finish primary school	−	−	−	−
Finished primary school	1.079^a^	0	1.04	1.12
Finished secondary school	1.181^a^	0	1.14	1.22
Finished high school	1.457^a^	0	1.40	1.51
University or graduate degree	1.991^a^	0	1.89	2.09
Maternal marital status				
Married at any time	−	−	−	−
Married	1.042	0.5	0.91	1.19
Single	1.168^a^	0.03	1.02	1.34
Maternal age				
< 17	−	−	−	−
18–24	1.039	0.1	1.00	1.08
25–34	1.356^a^	0	1.30	1.42
35–44	2.031^a^	0	1.92	2.15
≥ 45	2.288^a^	0	1.77	2.95
Maternal health affiliation program				
None	−	−	−	−
IMSS	1.323^a^	0	1.20	1.46
ISSSTE	1.384^a^	0	1.15	1.66
PEMEX, SEDENA, or SEMAR	1.570^a^	0.03	1.05	2.34
Seguro Popular	1.334^a^	0	1.28	1.39
Other	1.585^a^	0	1.31	1.91
IMSS-Oportunidades	1.089	0.1	1.00	1.19
Total number of prenatal checkups				
0–1	−	−	−	−
2–5	2.180^a^	0	1.99	2.39
≥ 6	2.593^a^	0	2.36	2.85
Trimester of first prenatal checkup				
None	−	−	−	−
First	0.572^a^	0	0.52	0.63
Second	0.517^a^	0	0.47	0.57
Third	0.483^a^	0	0.43	0.54
Newborn weight, g				
2500–3999	−	−	−	−
< 2500	1.271^a^	0	1.20	1.35
≥ 4000	1.880^a^	0	1.77	2.00
Gestational age, weeks				
37–41	−	−	−	−
< 28	0.458^a^	0	0.34	0.61
28–36	2.344^a^	0	2.20	2.49
42–45	2.510^a^	0	2.28	2.76
Facility where birth occurred				
Secretary of Health	−	−	−	−
IMSS-Oportunidades	1.163^a^	0	1.11	1.22
IMSS	1.268^a^	0	1.14	1.40
ISSSTE	2.218^a^	0	1.82	2.70
PEMEX, SEDENA, or SEMAR	0.920	0.7	0.61	1.39
Other public facility	2.864^a^	0	2.36	3.47
Private medical facility	10.131^a^	0	9.58	10.72
Indigenous presence				
Population with indigenous presence	−	−	−	−
Indigenous presence	1.407^a^	0	1.32	1.50
Dispersed indigenous population	0.690^a^	0	0.65	0.73
Community Human Development Index				
Low	−	−	−	−
Middle low	1.722^a^	0.01	1.17	2.54
Middle high	5.805^a^	0	3.94	8.56
High	4.786^a^	0	3.23	7.08
Multiple pregnancy				
No	−	−	−	−
Yes	4.329^a^	0	3.91	4.79
Hospitalization (days)				
0–1	−	−	−	−
2–7	1.250^a^	0	1.21	1.29
8–30	1.104^a^	0	1.07	1.14
31–99	1.016	0.4	0.98	1.05
≥ 100	1.078^a^	0	1.03	1.12

^a^The estimate is significant at 0.05

*IMSS* Instituto Mexicano del Seguro Social, *ISSSTE* Instituto de Seguridad y Servicios Sociales para los Trabajadores del Estado, *PEMEX* Servicios de Salud de Petróleos Mexicanos, *SEDENA* Servicios de Salud de la Secretaría de la Defensa Nacional, *SEMAR* Servicios de Salud de la Secretaría de Marina-Armada de México, *IMSS-Oportunidades* Instituto Mexicano del Seguro Social – Oportunidades (now known as Prospera)

Source: Prepared by the authors with information from DGIS, Subsystem of Information on Births

The C-section probability increased with the mother’s total amount of schooling. However, using a reference group of women with no schooling or who did not finish primary school, the difference for mothers who finished secondary school was very small. If a woman finished high school, the probability of having a C-section was 46% higher than for women in the reference group. The highest C-section probability was among women with the most schooling, i.e., college graduates or women involved in graduate studies; these women had an overall C-section rate that was 99% higher than for the reference group. The possibility of having a C-section was 17% higher for single mothers than for mothers married at any time.

The results showed that women <17-years-old were 4% more likely to have a C-section than 18–24-year-olds; for women 25–34-years-old, the probability increased to 36% and was double the rate for women ≥35-years-old, compared with women <17-years-old. The association between the mother’s age and schooling was reviewed in order to discard correlation. For this purpose, a model considering the interaction two variables was run. The model shows no variation in coefficients greater than 10% and most of the interaction coefficients were not significant.

If we exclude women affiliated with IMSS-Oportunidades (Prospera), whose probability of having C-sections is only 8% higher than for the reference group (no affiliation), women in the other categories had 32–58% higher probabilities of having C-sections. Women affiliated with IMSS were 1.32 times more likely to undergo C-sections than those without any healthcare affiliation; the results are similar for those affiliated with ISSSTE (1.38) and SP (1.33). In contrast, women affiliated with PEMEX, SEDENA, SEMAR, and other programs were approximately 58% more likely to have C-sections than women without a heath care affiliation. We also estimated that the possibility of having C-sections doubles for woman having 2–5 prenatal checkups and more than doubles for those having ≥6 checkups, compared with women having 0–1 checkups.

However, women who began their checkups during the first trimester had a 43% lower possibility of having a cesarean delivery than those without prenatal checkups; women who began their prenatal checkups during the second or third trimester had a much lower risk (49% and 52%, respectively) than those who did not have checkups.

Women who received care in IMSS-Oportunidades facilities had a 63% greater C-section possibility, compared with women receiving care at SSA; and IMSS are 26% higher than for women receiving care at SSA facilities (reference group). Women delivering at ISSSTE facilities had a 2.22-times higher C-section possibility than the reference group; women who gave birth at PEMEX, SEDENA, or SEMAR facilities had lower C-section possibilities than those in the SSA reference group. Women delivering in a private medical facility were 10 times more likely to undergo C-section than women attending an SSA hospital.

Women residing in municipalities with an indigenous population had a 40% higher C-section risk than those residing in municipalities with an indigenous presence. Residence in a municipality with a dispersed indigenous population was a protection factor (odds ratio, 0.69), with respect to cesarean deliveries.

Finally, the results of the model also suggest a direct relationship between the HDI and the frequency of C-sections. Compared with the reference group (low-HDI municipalities), the C-section possibility increased by 72% in communities with a medium-low HDI. Further, there was a 5.8 times greater chance of cesarean delivery for women residing in municipalities with a medium-high HDI and a 4.79 times greater chance in high-HDI municipalities.

## Discussion

In this study, we not only confirmed the findings reported in previous publications [4, 12, 13, 16, 44], but also offered additional insights on the factors associated to the increase of C-sections. Some variables that produced categorical results are related with the type of facility where the delivery occurred. For example, a cesarean birth is more likely to occur in private medical facilities [36–38] than in public facilities. Another variable is the direct relationship between C-sections and the mother’s level of schooling, as previous studies have shown [41, 44–50]. Whilst it could be thought that maternal age and schooling are both associated, in Chiapas this does not occur owing to the women education lag. Of the total number of women over 15 years of age in Chiapas, 36.2% are illiterate and 77.1% have incomplete basic education [51]. Therefore, it can be said that if schooling is taken into account, regardless of age, women with professional or postgraduate studies are more likely to have a cesarean delivery.

Our results indicate that single women are 17% more likely to have C-sections than those in the reference group. Among these women, 55% were <24-years-old and 12% were <18-years-old. Similarly, 87% were primigravida or secundigravida. These results coincide with those of a study carried out among Peruvian adolescents undergoing C-sections, with a similar demographic profile. Specifically, 62% were single and 82.1% were primigravida [52]. Therefore, the possibility of having a C-section increases when a woman is single, <24-years-old, and primigravida. These factors may highlight the vulnerability of single women to cesarean delivery.

An important finding of this study is the inclusion of structural variables, such as HDIs and SIL municipalities. When women had attained higher educational levels and socioeconomic development, they were more likely to undergo C-sections than women who were more disadvantaged (as previous studies have shown [41, 44–46]).

Contrary to previous reports [19, 53, 54], more C-sections occurred in municipalities with indigenous populations, suggesting that an indigenous woman delivering at a healthcare institution has a greater possibility of undergoing a cesarean delivery. For decades, international organizations have emphasized the lag in healthcare access among indigenous populations, and the need for countries to increase their coverage of these populations [54–56]. Therefore, we are interested in deepening on the strategies designed to expand this coverage, and the attendant effects, to indigenous women.

The Mexican government’s goal of reducing maternal mortality (MM) has been linked to the medicalization of childbirth. One strategy involved centralizing healthcare in states with larger indigenous populations [26, 57]. A nationwide study revealed a rate of maternal mortality (RMM) of 85 in municipalities with ≥70% SIL, which is more than twice the RMM of 37 in municipalities with <40% SIL [58]. Based on these results, we surmise that hospitals may be performing cesarean deliveries to avoid turning away patients who do not arrive in active labor and who, having been rejected, do not return. Early arrival or early labor may be a C-section risk factor (in primigravida patients <3 cm dilated and multigesta 6 cm) [59].

In 2016, the Committee to Promote Safe and Voluntary Maternity in Chiapas surveyed the opinions of 1986 women (381 expectant mothers and 1605 mothers with children <3-years-old) in towns with >80% SIL in the Chiapas region of Los Altos and in the city of San Cristóbal de Las Casas. The study aimed to evaluate the perinatal care preferences of indigenous women. The results indicated that only 25% of this population preferred hospital care. The rest of these women preferred care offered by midwives, relatives, or healthcare personnel in their own communities. However, this database mainly included indigenous women who were enrolled in the public health program because they received monetary transfers. These programs force women to change their maternity care practices [60].

The conditional cash transfer program in Mexico has increased C-section rates among the rural and poor sector; women who are considered to be high-risk are sent to a second level of care and are scheduled for C-sections with the effect of increases in the disposable income of the cash transfer.

Similarly, the fact that 40% of first or second births are cesarean deliveries may reflect a new strategy to resolve overcrowding in public obstetric/gynecology facilities in Chiapas. This suggests that childbirth medicalization is linked to the increased C-section rate in this area.

We also found a positive relationship between the number of prenatal checkups and the C-section rate. The relationship between the number of prenatal checkups and the increased numbers of C-sections has also been documented by other authors [44, 45, 61]. These authors noted that insurers provided higher rates of remuneration to doctors with patients undergoing checkups; doctors justify these remunerative actions by the greater preponderance of checkups. Although SP insures 49.9% [51] of the Mexican people, and pays twice as much for a cesarean birth as it does for a vaginal birth, an additional payment for checkups is not made by this public health institution [4]. Private insurances cover only a small percentage of the Chiapas population; hence, they cannot fully explain this close relationship. Thus, the current policies aimed at reducing MM may have led to an intentional selection of high-risk women and an increased number of C-sections.

An inverse relationship was found between the possibility of a C-section and the later onset of prenatal checkups; specifically, when women had their first checkup during the third trimester of pregnancy, they were less likely to undergo cesarean delivery. However, the possibility of a C-section was higher when women did not receive any prenatal care. Women who do not attend prenatal care are likely to only go to the hospital if they feel they are experiencing complications. This is consistent with an investigation conducted in Chihuahua, Mexico, among pregnant adolescents. In that study, 34% of 19-year-old indigenous women, as opposed to 20% of similarly aged non-indigenous women, underwent C-sections following indications of preeclampsia and fetal distress. These complications were attributed to a lack of prenatal care [62]. The indigenous population in Mexico is highly impoverished and marginalized [63]. Reflecting this, indigenous women in Chiapas commonly begin bearing children early in life, and many deliver their children at home [64, 65]; thus, some of these births are likely not reflected in the available statistics.

C-section rates in Chiapas, and more generally throughout Mexico, are much higher than the expected standards. Governmental interest in reducing the MM has likely contributed to this observation. However, in 2002, a study was conducted to examine representative C-section data, obtained from surveys and administrative records, from 129 countries worldwide [66]. That study investigated the relationship between cesarean delivery and MM rates, based on a non-parametric regression method. The estimated average rate of C-sections was 15%. Comparing this rate with country-specific MM (RMM), a clear relationship is not observed, even when there may be a correlation between higher C-section rates and lower RMM [4]. In Latin America and the Caribbean, C-section rates were higher than in other developing countries, but lower than the average C-section rates in developed countries [66]. Inverse relationships between cesarean delivery rates and maternal and fetal mortality rates were observed in poor countries that provide little access to obstetric care. Furthermore, C-section rates above certain threshold values do not translate into higher maternal or neonatal benefits, and may lead to negative consequences [67].

Our results show that cesarean deliveries are also related to other factors. For example, educated women and those with a higher quality of life are more likely to have C-sections as they believe that this method is the safest, easiest, and quickest way to give birth, and that vaginal births are old-fashioned [68, 69]. Thus, for this population, the main reason for having C-sections is the ability to schedule an exact date, allowing the father and other relatives to be present. The care of older offspring can also be scheduled, and painful labor, often lasting 6–10 h, can be avoided. This has been further enabled by new technologies that, besides informing parents of their baby’s sex so they may choose an appropriate name, can establish the degree of fetal maturity and make it easier to select an optimal delivery date. These justifications are also strengthened by the practices of healthcare professionals [70–73].

Conversely, more impoverished women and those with lower quality lives have fewer delivery possibilities and are rarely informed about the risks associated with C-sections. Thus, certain rights advocates believe that when a C-section is unnecessary and unjustified, the procedure may constitute a violation of the woman’s reproductive rights. The Mexican government has admitted partial or total failure in its duty to “respect, protect, and guarantee human rights, of which women’s rights are a part” ([74] p. 20). Chiapas and Mexico City are the only subnational entities that include reproductive rights in their Laws of Women’s Access to a Life Free of Violence. Moreover, unjustified C-section is considered violence in the women’s Life Free of Violence laws of nine Mexican states and only in Chiapas^15^ and Veracruz^16^ it forms part of the “obstetric violence” crime specified in its criminl code.

Within medical associations [75], it has been acknowledged that the performing of elective C-sections is due to their working conditions and, thus, places the responsibility for the violation of women’s rights on the Mexican state. However, a statement from the Observatory of Maternal Mortality in Mexico [76] indicated that this stance is dubious, as most cesarean births (as compared with vaginal births) occur in private medical practices (86% in Chiapas and 80% nationally, in 2014),^17^ where private specialists could, in fact, accompany the process of labor and birth. So, a call upon the government is made to improve infrastructure, training, and human resources for providing holistic care following childbirth.

When the need for a C-section is clearly justified, physicians can save lives; however, the procedure is more costly for institutions, owing to the required inputs and medical equipment, and to the general public, owing to the short-, medium-, and long-term complications of the procedure. Women undergoing C-sections have a 25% higher possibility of remaining hospitalized for 2–7 days, a 10% higher possibility of remaining hospitalized for 8–30 days, and an 8% higher probability of remaining hospitalized for ≥100 days. These numbers point to the higher risk of complications.

Furthermore, since the establishment of private medical insurance companies in Mexico, the number of C-sections has grown exponentially, clearly indicating a cost-benefit relationship. A similar situation exists in public facilities. Since 2004, the SP has, together with other medical insurance plans, paid operating facilities twice the amount for a C-section as for a vaginal delivery. In addition, some services covered by the SP can be subcontracted to the private sector [77]. In Chiapas, the poorest families are signing up for this policy [78], which means that low-income women are exempt from paying for obstetric care, regardless of the delivery procedure required.

Careful study should be undertaken to investigate how these new health policies impact the number of C-sections in public hospitals in Mexico. Such policies may lead many women to submit to unnecessary C-sections, even if they lack the higher-risk conditions that have historically driven the need for C-sections.

This study underscores the need for a variety of actions, as unjustifiable C-sections are a multicausal problem. Clearly, institutional organization and the regulation of medical personnel limit adherence to institutional protocols and federal and state laws. We consider that, in the case of indigenous populations, strengthening primary care by hiring professional midwifes to attend vaginal deliveries is important. This is a strategy that the Mexican government and the state of Chiapas, specifically, must strengthen. We have previously made specific recommendations for the reduction of maternal mortality [79, 80], and will not reiterate those here.

This study has some limitations, including a paucity of the detailed information necessary to address the interrelationship between the fundamental factors driving cesarean deliveries, such as the number of women who request cesarean delivery, how birth order influences C-section rates, or the fact that the absence of prenatal care increases the C-section probability. The dynamics leading to the observed increase in C-sections among indigenous women should be elucidated. Little information is available, even in the administrative records of hospital discharges from public institutions, regarding physician rationales for performing C-sections. Such a study would require an analysis of the clinical files of indigenous women, in particular, who are unlikely to be available to an outside researcher. Therefore, this analysis was not included in the study; such decisions or specificities cannot be identified or taken from the existing database, and would require more extensive fieldwork and archiving.

In the future, the SINAC database will allow us to conduct a longer-term analysis based on reliable information for estimating the tendency of C-sections, and thus, in a context of sexennial public policies, to compare Mexican government’s efforts to reduce C-sections.

## Conclusions

We identified some of the factors associated with the increased numbers of C-sections in Chiapas, Mexico. The study considered women who reside in municipalities with indigenous populations and municipalities with higher HDIs, and among educated women and those undergoing regular prenatal checkups. Although C-sections are associated with women of middle or high socioeconomic status, the most important finding of this study is that the increase in the numbers of C-sections among indigenous women in Chiapas appears to be related to policies aimed at reducing MM. Furthermore, the analysis showed an increased C-section rate among women giving birth for the first or second time. The current results, in the context the recent legislative changes, allow us to conclude that the medicalization of childbirth and the increased C-section rate represent obstetric violence. Owing to the health risks associated with C-sections, we stress the need to implement actions to limit the number of C-sections. Better institutional organization is required to create space for women to recuperate. Medical regulations are needed to ensure that protocols and technical norms are being adequately implemented. Awareness should be increased among health personnel and the public regarding women reproductive rights. To this end, actions should be taken to limit or prevent procedures that are considered obstetric violence, including, where appropriate, exposing them to the public. In the particular case of reducing C-sections among indigenous populations, strengthening primary healthcare and promoting professional midwife attendance at normal births are necessary.

## Abbreviations

DGIS: General direction of health information
DNA: Deoxyribonucleic acid
FEMECOG: Mexican federation of colleges of obstetrics and gynecology
HDI: Human development index
IMSS: Instituto Mexicano del Seguro Social
IMSS-Oportunidades: Instituto Mexicano del Seguro Social – Oportunidades (now known as Prospera)
ISSSTE: Instituto de Seguridad y Servicios Sociales para los Trabajadores del Estado
MM: Maternal mortality
PEMEX: Servicios de Salud de Petróleos Mexicanos
RMM: Rate of maternal mortality
SEDENA: Servicios de Salud de la Secretaría de la Defensa Nacional
SEMAR: Servicios de Salud de la Secretaría de Marina-Armada de México
SIL: Speakers of indigenous languages
SP: Seguro popular
SSA: Secretary of health
UNDP: United Nations development program

## Footnotes

1 Williams JW. A criticism of certain tendencies in American obstetrics. NY State J Med. 1922;22:493-9, quoted in Cyr RM. Myth of the ideal cesarean section rate: commentary and historic perspective. Am J Obstet Gynecol. 2006;194:933.

2 General Direction of Health Information (DGIS). Database of registered live births INEGI 1990-2013 [online]. National Health Information System (SINAIS).

3 Information from the General Census of Population and Housing, 2000.

4 Information from the Census of Population and Housing, 2005.

5 Calculations based on information from the DGIS/SSA bulletins, 2015.

6 The HDI has three aspects: health, income, and education. “In health, the rate of infant survival is the basis for approximating the information that life expectancy at birth would provide. In the Education Index, the average years of schooling and the expected years of schooling are used to compile the combined education index. As a proxy for income, the municipal per-capita income is considered and adjusted to the annual Gross National Income (GNI) in U.S. dollars, adjusted for purchasing power parity (PPP). The three indexes are added by using the geometric mean and the result is the HDI, expressed in values between zero and one, where one denotes the best possible achievement and zero indicates no advance” (28, p. 14).

7 “Married” can also refer to a non-formal situation such as cohabitation.

8 Mexican Social Security Institute.

9 Institute for Security and Social Services for Workers of the State.

10 Health Services of Petróleos Mexicanos.

11 Health Services of the Secretary of National Defense and the Secretary of the Navy.

12 Popular [Health] Insurance.

13 Mexican Social Security Institute – Oportunidades, now known as Prospera.

14 Secretary of Health.

15 Article 183 Ter of the Penal Code of Chiapas.

16 Article 363 of the Penal Code of Veracruz.

17 Information from SINAC/SINAIS/DGIS/SSA, Cubos, México, 2015.

## References

[1] Betran AP, Torloni MR, Zhang J (2015). What is the optimal rate of caesarean section at population level? A systematic review of ecologic studies. Reprod Health.

[2] Eckerlund I, Gerdtham UG (1999). Estimating the effect of cesarean section rate on health outcome, Evidence from Swedish Hospital Data. Int J Technol Assess Health Care.

[3] Berghella V, Baxter JK, Chauhan SP (2005). Evidence-based surgery for cesarean delivery. Am J Obstet Gynecol.

[4] Cárdenas R, Sánchez B (2014). El perfil de utilización de la cesárea en México y su implicación para la salud reproductiva. Desigualdades en la procreación. Trayectorias reproductivas, atención obstétrica y morbimortalidad materna en México.

[5] Organización Mundial de la Salud (OMS), Human Reproductive Program (HRP). Declaración de la OMS sobre tasas de cesárea. Resumen ejecutivo. Ginebra: WHO/RHR, 2015. Available at: http://apps.who.int/iris/bitstream/10665/161444/1/WHO_RHR_15.02_spa.pdf [cited 28 June 2015].

[6] Porreco R, Thorp J (1996). The cesarean birth epidemic: trends, causes, and solutions. Am J Obstet Gynecol.

[7] Hofmeyr J, Novikova N, Mathai M, Shah A (2009). Techniques for cesarean section. Am J Obstet Gynecol.

[8] Boyle A, Reddy U (2012). Epidemiology of cesarean delivery: the scope of the problem. Sem Perinatol.

[9] Weaver J, Statham H, Richards M (2007). Are there “unnecessary” cesarean sections? Perceptions of women and obstetricians about cesarean sections for nonclinical indications. Birth.

[10] ENSANUT (2012). (Encuesta Nacional de Salud y Nutrición). Documentos analíticos. Elevada recurrencia a las cesáreas: revertir la tendencia y mejorar la calidad en el parto.

[11] DGIS/SSA. Boletín de Información Estadística 2000-2011, volume III, 2015. Available at: http://www.dgis.salud.gob.mx/contenidos/publicaciones/p_bie.html. Accessed 22 Jul 2015.

[12] Puentes E y cols (2004). Las cesáreas en México: tendencias, niveles y factores asociados. Salud Publica Mex.

[13] Suárez L (2013). Características sociodemográficas y reproductivas asociadas con el aumento de cesáreas en México. Salud Publica Mex.

[14] Martínez GJ (2015). Operación cesárea. Una visión histórica, epidemiológica y ética para disminuir su incidencia. Rev Med Inst Mex Seguro Soc.

[15] Vallejos A (2011). Tendencias y factores asociados a cesáreas en México: validación de un instrumento. Perinatol Reprod Hum.

[16] Rosado J (2013). Frecuencia, Indicaciones y conocimiento de embarazadas sobre la cesárea: el caso de un hospital de la ciudad de Mérida, Yucatán. Rev Biomed.

[17] Juárez C (1999). Tendencia de los embarazos terminados por operación cesárea en México durante el periodo 1991-1995. Ginecol Obstet Mex.

[18] INSP (2009). Evaluación externa del programa IMSS Oportunidades para el ejercicio fiscal 2008.

[19] Nazar A (2007). Atención del parto, migración rural-urbana y políticas públicas de salud reproductiva en población indígena de Chiapas. Ra Ximhai.

[20] La WM (2011). Etnia y las cesáreas en los partos en el hospital público de Arica.

[21] Secretaría de Hacienda. Cuarto Informe de Gobierno. http://www.chiapas.gob.mx/media/informes/2008-2012/4-Informe-2010/anexo-1/Anexo-I-Indicadores.pdf (2010). Accessed 28 Jul 2015.

[22] Secretaría de Planeación, Gestión Pública y Programa de Gobierno. Segundo Informe de Gobierno. Available at: http://www.chiapas.gob.mx/informe/(2010). Accessed 19 Aug 2015.

[23] Gómez-Dantés O (2004). El secuestro de Lucina (o cómo detener la epidemia de cesáreas). Salud Publica Mex.

[24] Araujo FJ, Encinas C, Araujo FJ, Torres MA, Caballero MV. Asepsia y Antisepsia. Visión histórica desde un cuadro. Apuntes de Ciencia -– Boletín Científico HGUCR; 2011:60-64. http://apuntes.hgucr.es/2011/06/27/asepsia-y-antisepsia-vision-historica-desde-un-cuadro/(2011) Acceced on 10 Aug 2015.

[25] Becerro de Bengoa C (2001). Analgesia y anestesia en obstetricia. Toko-Gin Pract.

[26] Betrán A, Merialdi M, Lauer J (2007). Rates of caesarean section: analysis of global, regional and national estimates. Paediatr Perinat Epidemiol.

[27] Villar J, Valladares E, Wojdyla D (2006). Caesarean delivery rates and pregnancy outcomes: the 2005 WHO global survey on maternal and perinatal health in Latin America. Lancet.

[28] Belloso WH (2009). Historia de los antibióticos. Rev Hosp Ital B Aires Dic.

[29] Henderson E, Love EJ (1995). Incidence of hospital-acquired infections associated with caesarean section. J Hosp Infect.

[30] Rosales E, Felguérez JA (2009). Repercusión demográfica de la operación cesárea. Ginecol Obstet Mex.

[31] Joswik M, Joswik M, Lotocki W (1997). Vesicouterine fistula – an analysis of 24 cases from Poland. Int J Gynaecol Obstet.

[32] Cárdenas R (2002). Complicaciones asociadas a la cesárea: la importancia de un uso módicamente justificado. Gac Med Mex.

[33] Secretaría de Salud (2009). Diagnóstico y Tratamiento de la Hemorragia Obstétrica en la Segunda Mitad del Embarazo y Puerperio Inmediato.

[34] Instituto Mexicano de Seguro social. Guía de Práctica Clínica para la Reducción de la Frecuencia de Operación Cesárea México. México: 2014. http://www.cenetec.salud.gob.mx/descargas/gpc/CatalogoMaestro/048_GPC_Cesarea/IMSS_048_08_EyR.pdf (2014) Acceced on 8 Sept 2016.

[35] Almgren M, Schlinzig T, Gomez D (2011). Cesarean delivery and hematopoietic stem cell epigenetics in the newborn infant: implications for future health?. Am J Obstet Gynecol.

[36] Cho CE, Norman M (2013). Cesarean section and development of the immune system in the offspring. Am J Obstet Gynecol.

[37] Freyermuth MG, Luna M (2014). Muerte materna y muertes evitables en exceso. Propuesta metodológica para evaluar la política pública en salud. Realidad, datos y espacio. Revista Internacional de Estadística y Geografía.

[38] Gibbons L, Belizan JM, Lauer J (2012). Inequities in the use of cesarean section deliveries in the world. Am J Obstet Gynecol.

[39] PNUD. Índice de Desarrollo Humano Municipal en México. http://www.mx.undp.org/content/dam/mexico/docs/Publicaciones/PublicacionesReduccionPobreza/InformesDesarrolloHumano/UNDPMX-PovRed-IDHmunicipalMexico-032014.pdf (2014) Accessed 30 Jul 2015.

[40] Dirección General de Información en Salud (DGIS). Base de datos de Certificado de Nacimiento – Nacimientos ocurridos, 2013 [online]. Sistema Nacional de Información en Salud (SINAIS). Available at: http://www.dgis.salud.gob.mx/contenidos/basesdedatos/certnac_sinac13.html (2015) Accessed 22 Apr 2015.

[41] INEGI. Censo de Población y Vivienda 2010. Principales resultados por localidad (ITER), 2013. Available at: http://www.inegi.org.mx/sistemas/consulta_resultados/iter2010.aspx (2010). Accessed 28 June 2015.

[42] Hilbe J (2009). Logistic regression models.

[43] Boado H (2013). Introducción al análisis multinivel.

[44] Gould JB, Davey B, Stafford RS (1989). Socioeconomic differences in rates of cesarean section. N Engl J Med.

[45] Soto-Vega E, et al. The epidemic of the cesarean section in private hospital in Puebla, México. Obstet Gynecol Int J. 2015;2(6):4-5.

[46] Gómez U (1999). Risks factors for the increasing cesarean section rate in Southeast Brazil: a comparison of two births cohort, 1978-1979 and 1994. Int J Epidemiol.

[47] El FG (2015). subsistema de información sobre nacimientos. Estudio de caso en la región tsotsil-tseltal de Chiapas, México. Ponencia de Jornadas académicas.

[48] Buttino I, Tozzi L, Bocciolone L, Pírotta N, Prazzini F (1990). Determinantes de la frecuencia de cesárea en Italia, 1980-1983. Ann Obstet Ginecol Med Perinat.

[49] Parazzini F, Pirotta N, LaVecchia C, Fedele L (1992). Determinants of caesarean section rates in Italy. Br J Obstet Gynaecol.

[50] Taffel SM. Cesarean delivery in the United States, 1990. Vital & Health Statistics. Series 21. Data on Natality, Marriage & Divorce. 1994:1-24. 8049307

[51] Instituto Nacional de Estadística y Geografía (INEGI). Principales resultados de la Encuesta Intercensal 2015. Estados Unidos Mexicanos. México: INEGI; 2016.

[52] Albornoz A, y Reártegui N. Cesáreas en adolescentes atendidas en el Hospital Nacional Santa Rosa durante el periodo 2010 – 2012 para optar el Título Profesional de Licenciado en Obstetricia. Universidad Nacional Mayor de San Marcos, Facultad de Medicina Humana, Lima Perú, 2013.

[53] Sánchez-Pérez HJ, Vargas Morales G, Jansá JM. Vida y salud de la mujer en zonas de alta marginación en México. Es peor ser indígena? En publicación: Pueblos indígenas y pobreza. Enfoques multidisciplinarios. Cimadamore, Alberto D.; Eversole, Robyn; McNeish, John-Andrew. Programa CLACSO-CROP, Argentina, 2006.

[54] Mahoney S, Malcoe L (2005). Cesarean delivery in Native American women: are low rates explained by practices common to the Indian health service?. Birth.

[55] Organización Panamericana de la salud/Organización Mundial de la Salud (OPS/OMS). Iniciativa de Salud de los Pueblos Indígenas. Organización Panamericana de la Salud/Organización Mundial de la Salud OPS/OMS. http://iris.paho.org/xmlui/bitstream/handle/123456789/7015/15688.pdf (1993). Accessed 18 Dec 2015.

[56] Comisión Económica para América Latina (CEPAL). Primera reunión de la Conferencia Regional sobre Población y Desarrollo de América Latina y el Caribe. Integración plena de la población y su dinámica en el desarrollo sostenible con igualdad y enfoque de derechos: clave para el Programa de Acción de El Cairo después de 2014 Montevideo, 12 a 15 de agosto de 2013. CEPAL-Naciones Unidas. http://www.acnur.org/t3/fileadmin/Documentos/BDL/2013/9232.pdf?view=1 (2013). Accessed 23 Sept 2016.

[57] Secretaría de Salud/Centro Nacional de Equidad de Género y Salud Reproductiva. Estrategia Integral para Acelerar la Reducción de la Mortalidad Materna en México. http://www.coneval.gob.mx/rw/resource/coneval/info_public/Estrategia_Integral.pdf (2009) Accessed 3 Feb 2017.

[58] Freyermuth G (2016). Determinantes sociales en la Mortalidad Materna en México. Revista CONAMED.

[59] Holmes P, Oppenheimer L, Wen SW (2001). The relationship between cervical dilatation at initial presentation in labour and subsequent intervention. BJOG.

[60] Barber S (2010). Mexico’s conditional cash transfer programme increases cesarean section rates among the rural poor. Eur J Public Health.

[61] Braveman P (1995). Racial/ethnic differences in the likelihood of cesarean delivery, California. Am J Public Health.

[62] Monarrez-Espino J (2009). Salud y nutrición en adolescentes tarahumaras. Rev Med Inst Mex Seguro Soc.

[63] Consejo Nacional de Evaluación de la Política Social (CONEVAL). Resultados de pobreza en México 2014 a nivel nacional y por entidades federativas. http://www.coneval.gob.mx/Medicion/MP/Paginas/Pobreza_2014.aspx (2015) Accessed 8 Aug 2015.

[64] Moreno Guati-Rojo M, Rubio Blanco C, Sánchez Ramírez G (2015). Caracterización de la partería en 6 municipios de los Altos de Chiapas: Retos y desafíos de su ejercicio en contextos actual. Imagen Instantánea de la Partería.

[65] Nazar Beutelspecher A, Salvatierra Izaba B, Morales Domínguez M, Hartman A, Rodríguez Mazariegos R. Estudio cualitativo de barreras de demanda y oferta con enfoque a nivel local y comunitario y cambio de comportamiento en municipios prioritarios de Chiapas, México: Informe de las Regiones Altos Tseltal-Tsotsil y Tulijá Tseltal-Chol para el Banco Interamericano de Desarrollo. http://idbdocs.iadb.org/wsdocs/getdocument.aspx?docnum=38904122 (2011) Accessed 4 Apr 2016.

[66] Gapminder. Razón de mortalidad materna en el periodo 1980 a 2007. http://www.gapminder.org/Accessed 20 Aug 2015.

[67] Belizán JM (2007). Health consequences of the increasing caesarean section rates. Epidemiology.

[68] Gonzalez G, Vega M, Cabrera C, Muñoz A, Valle A (2001). Caesarean sections in Mexico: are there too many?. Health Policy Plan.

[69] Penna L, Arulkumaran S (2003). Cesarean section for non-medical reasons. Int J Gynaecol Obstet.

[70] Plante L (2006). Public health implications of cesarean on demand. Obstet Gynecol Surv.

[71] Farland L (2009). The use and overuse of cesarean sections in Mexico. TuftScope.

[72] Villanueva L (2004). Operación cesárea: una perspectiva integral. Rev Fac Med UNAM.

[73] Queenan JT (2004). Elective cesarean delivery. Obstet Gynecol.

[74] Facio A (2008). Necesidad de enmarcar la salud sexual y reproductiva en un marco de derechos humanos. Los derechos reproductivos son derechos humanos.

[75] FEMECOG. Pronunciamiento. https://drive.google.com/file/d/0B_Wsl17nCOpWd0d0d3ZFUnNEdUk/view (2015). Accessed 2 Aug 2015.

[76] OMM. Posicionamiento del Observatorio de Mortalidad Materna frente al Pronunciamiento de la Federación Mexicana de Colegios de Obstetricia y Ginecología, A.C. en relación con la violencia obstétrica. www.omm.org.mx/images/stories/Documentos%20grandes/Posicion_del_OMM_FEMECOG.docx (2015) Accessed 26 Aug 2015.

[77] Leal G (2013). ¿Protección social en salud? Ni “seguro”, ni “popular”. Estudios Políticos.

[78] Meneses S, Echeverría D (2007). Acceso universal a la atención obstétrica? El Seguro Popular de Salud frente al reto de la muerte materna en Los Altos de Chiapas. Muerte materna y seguro popular.

[79] Freyermuth G (2012). Evaluación Estratégica sobre Mortalidad Materna en México 2010: características sociodemográficas que obstaculizan a las mujeres embarazadas su acceso efectivo a instituciones de salud.

[80] Secretaría de Salud. Convenio General de Colaboración que celebran la SSA, el IMSS y el ISSSTE para la Atención de las Emergencias Obstétricas http://www.omm.org.mx/images/stories/Documentos%20grandes/ceo_conv.pdf (2009). Accessed 28 May 2009.

